# Evaluation of Computer-Aided Nodule Assessment and Risk Yield (CANARY) in Korean patients for prediction of invasiveness of ground-glass opacity nodule

**DOI:** 10.1371/journal.pone.0253204

**Published:** 2021-06-14

**Authors:** Juyoung Lee, Brian Bartholmai, Tobias Peikert, Jaehee Chun, Hojin Kim, Jin Sung Kim, Seong Yong Park

**Affiliations:** 1 Department of Radiation Oncology, Yonsei Cancer Center, Yonsei University College of Medicine, Seoul, Korea; 2 Department of Integrative Medicine, Major in Digital Healthcare, Yonsei University College of Medicine, Seoul, Korea; 3 Department of Radiology, Mayo Clinic, Rochester, Minnesota, United States of America; 4 Division of Pulmonary and Critical Care Medicine, Department of Medicine, Mayo Clinic, Rochester, Minnesota, United States of America; 5 Department of Thoracic and Cardiovascular Surgery, Yonsei University College of Medicine, Seoul, Korea; Vellore Institute of Technology: VIT University, INDIA

## Abstract

Differentiating the invasiveness of ground-glass nodules (GGN) is clinically important, and several institutions have attempted to develop their own solutions by using computed tomography images. The purpose of this study is to evaluate Computer-Aided Analysis of Risk Yield (CANARY), a validated virtual biopsy and risk-stratification machine-learning tool for lung adenocarcinomas, in a Korean patient population. To this end, a total of 380 GGNs from 360 patients who underwent pulmonary resection in a single institution were reviewed. Based on the Score Indicative of Lung Cancer Aggression (SILA), a quantitative indicator of CANARY analysis results, all of the GGNs were classified as “indolent” (atypical adenomatous hyperplasia, adenocarcinomas *in situ*, or minimally invasive adenocarcinoma) or “invasive” (invasive adenocarcinoma) and compared with the pathology reports. By considering the possibility of uneven class distribution, statistical analysis was performed on the 1) entire cohort and 2) randomly extracted six sets of class-balanced samples. For each trial, the optimal cutoff SILA was obtained from the receiver operating characteristic curve. The classification results were evaluated using several binary classification metrics. Of a total of 380 GGNs, the mean SILA for 65 (17.1%) indolent and 315 (82.9%) invasive lesions were 0.195±0.124 and 0.391±0.208 (*p* < 0.0001). The area under the curve (AUC) of each trial was 0.814 and 0.809, with an optimal threshold SILA of 0.229 for both. The macro F1-score and geometric mean were found to be 0.675 and 0.745 for the entire cohort, while both scored 0.741 in the class-equalized dataset. From these results, CANARY could be confirmed acceptable in classifying GGN for Korean patients after the cutoff SILA was calibrated. We found that adjusting the cutoff SILA is needed to use CANARY in other countries or races, and geometric mean could be more objective than F1-score or AUC in the binary classification of imbalanced data.

## Introduction

Lung cancer is one of the most common causes of cancer-related deaths throughout the world, and lung adenocarcinoma is the most common histologic subtype of lung cancer [1]. Recently, the incidence of early lung adenocarcinoma, which is mainly diagnosed by observing the presence of ground glass opacity nodules (GGNs) on chest computed tomography (CT), has been increasing due to the development of chest CT and surveillance programs. According to the 2011 International Association for the Study of Lung Cancer/American Thoracic Society/European Respiratory Society (IASLC,ATS,ERS) guidelines, lung adenocarcinomas can be classified as atypical adenomatous hyperplasia (AAH), adenocarcinoma *in situ* (AIS), minimally invasive adenocarcinoma (MIA), and invasive adenocarcinoma (IA), depending on the size of the lesion and the presence of invasive components on the pathological analysis [2]. These classifications are well correlated to survival rates; disease-free survival in early-stage AIS and MIA patients is close to 100% [3], while disease-free survival in IA patients is 60–70%. However, this discrimination is based on postoperative pathologic results, even though preoperative differentiation is crucial for establishing appropriate treatment plans and follow-up strategies.

Computer-Aided Nodule Assessment and Risk Yield (CANARY), which is based on a machine learning technique, is one of the most advanced types of software. It has been steadily improved, and its performance has been verified through previous studies [4–9]. CANARY divides semi-auto segmented GGN regions of interest into nine distinct exemplars based on radiomic features and clusters them into three separate groups for risk stratification. In particular, through the concept of Score Indicative of Lung Cancer Aggression (SILA), an accumulated sum of normalized distributions of CANARY exemplars, the optimal SILA cutoff was set to divide indolent and invasive retrospectively, based on the survival rate [9]. As mentioned above, the sub-classification of GGN is clinically meaningful, and CANARY is one of the leading tools used for this. However, CANARY has been verified mostly for datasets in the United States only, and its validity has rarely been verified for data of other countries or races, especially from the Asian cohort. Therefore, in this study, we evaluated the versatility and performance of CANARY in indolent-and-invasive separation by applying it to data collected from Korean patients and found that it provided reliable performance in distinguishing between indolent and invasive nodules from the chest CT images of the patients.

## Materials and methods

### Patients

This retrospective study was approved by the Institutional Review Board of Severance Hospital (IRB No. 4-2020-0856). Because this study was retrospective IRB waived the requirement for informed consent of patients. All data were fully anonymized. A total of 360 preoperative chest CT sets from patients who received pulmonary resection for GGN at Severance Hospital, Seoul, South Korea between January 2018 and July 2020 were retrospectively reviewed, and basic demographics and pathologic reports were collected from the electrical medical records. The 380 lesions were classified as indolent (atypical adenomatous hyperplasia (AAH), adenocarcinoma *in situ* (AIS), minimally invasive adenocarcinoma (MIA)) or invasive (invasive adenocarcinoma) based on the pathological reports.

### Algorithm of CANARY and application method

CANARY was developed using machine learning clustering algorithms from radiomic features of lesions, and the results of CANARY are represented via pie charts, overall prognostic characterization, and SILA. SILA is an accumulated sum of normalized distributions of CANARY exemplars, which allows the CANARY analysis results to be used as a quantitative indicator that has proven to have significant performance in distinguishing between indolent and invasive forms of adenocarcinoma [9]. The detailed algorithm and the order of use is published previously [4, 5, 7]. A board-certified thoracic surgeon (S Y Park) confirmed the location and area of each GGN on the CT image in CANARY. After acquiring the CANARY results for each GGN case, an exploratory analysis was conducted to determine the significance of SILA in distinguishing between indolent and invasive. The SILA distribution of each group was confirmed by using the median with an interquartile range.

### Statistical indicators to evaluate the performance of CANARY

The indexes mainly used when evaluating the performance of a binary classification model include area under the curve (AUC) from the receiver operating characteristic (ROC) curve and accuracy, sensitivity, specificity, and F1-score that could be derived from the confusion matrix. However, these indicators have limitations. Since AUC is a comprehensive indicator of performance according to all thresholds, it does not sufficiently reflect the clinical needs to use the optimal threshold and classify the GGNs according to certain criteria. More importantly, accuracy, sensitivity, specificity, and F1-score including AUC may not be objective if the class distribution is imbalanced. In addition, some indicators only consider true positive (TP) or true negative (TF), while the other side is excluded from the definition, which is not suitable for this study where the classification for both indolent and invasive should be evaluated. Accordingly, all indicators used in this study contain both TP and TN after normalized from 0 to 1 for intuitive comparison, meaning that 0 for the worst and 1 for the best result. The indicators used are summarized in Table 1.

**Table 1:** Evaluation indicators to evaluate and compare the performance of Computer-Aided Nodule Assessment and Risk Yield.

Indicators	Definition (Formula)	Range Interval worst ↔ best
Accuracy	$\frac{TP+TN}{TP+TN+FP+FN}$	[0, 1]
F1-score	$\frac{2\cdot TP}{2\cdot TP+FP+FN}$	[0, 1]
macro F1-score [11]	$\left(\frac{2\cdot TP}{2\cdot TP+FP+FN}+\frac{2\cdot TN}{2\cdot TN+FP+FN}\right)\;/\;2$	[0, 1]
Matthews correlation coefficient (MCC) [12]	$\frac{TP\cdot TN-FP\cdot FN}{\sqrt{\left(TP+FP)\cdot (TP+FN)\cdot (TN+FP)\cdot (TN+FN\right)}}$	[-1, 1]
*normalized* MCC (nMCC) [13, 14]	$\frac{MCC+1}{2}$	[0, 1]
Bookmaker Informedness (BM) [14]	$\frac{TP}{TP+FN}+\frac{TN}{FP+TN}-1$	[-1, 1]
*normalized* BM (nBM) [13]	$\frac{BM+1}{2}$	[0, 1]
Geometric mean(GM) [16]	$\sqrt{\frac{TP}{TP+FN}\cdot \frac{TN}{FP+TN}}$	[0, 1]

TP; True Positive, TN; True Negative, FP; False Positive, FN; False Negative.

### Analysis of CANARY result

First, by using CANARY results for the entire cohort, a ROC curve was plotted to evaluate the performance of CANARY and to find the optimal SILA of classifying indolent and invasive. Subsequently, an AUC was calculated from the ROC curve, and various metrics for binary classification–such as accuracy, macro F1-score, normalized Matthews correlation coefficient (nMCC), normalized Bookmaker Informedness (nBM), and geometric mean (GM)–were also derived and summarized (Table 1).

Next, the aforementioned evaluation process was conducted on the six groups of dataset with uniform distribution of indolent and invasive to prevent the potential overfitting problem due to class imbalance. The six equalized datasets were acquired by simple random sampling method applied to the majority class. In this case, after macro and micro AUC were obtained respectively, the confusion matrix for the optimal cutoff was similarly arranged to derive values for the same indicators as mentioned above.

**Fig 1:**
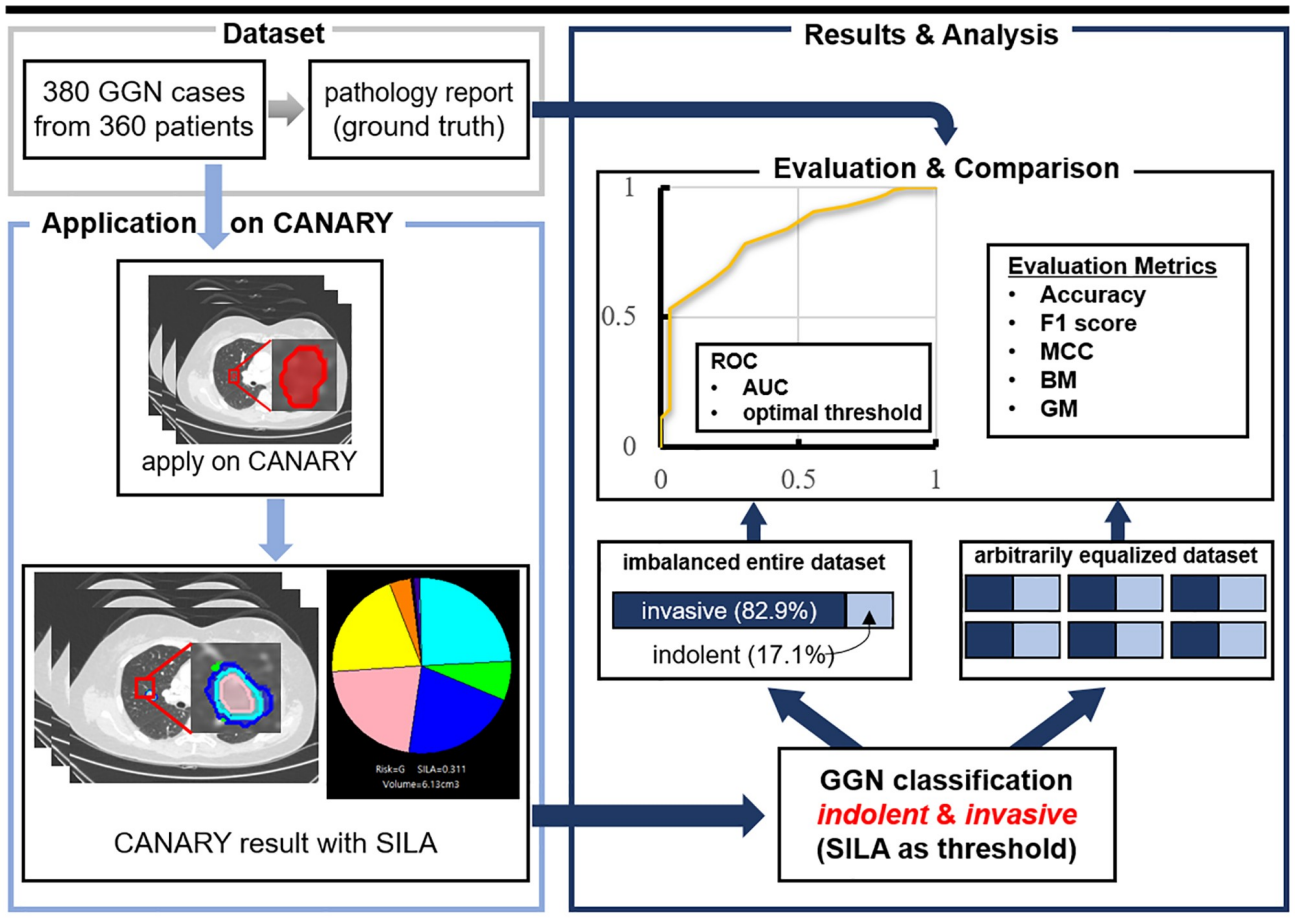
Flowchart of Computer-Aided Nodule Assessment and Risk Yield (CANARY) validation study. CT images from the 360 patients were applied on CANARY, and classification result analysis was performed on the entire dataset and the uniformly resampled dataset, respectively. Then, the quantitative scores were checked by using evaluation indicators and compared with the scores from the confirmed pathology report.

Lastly, the results of each step and item are compared with the corresponding item in the previous studies from Mayo Clinic. According to the characteristics of the item, a Mann-Whitney test was conducted to confirm the statistical significance between this study and the previous studies. The overall concept of this study is presented in Fig 1. Python 3.7.6 and R 4.0.2 were used for data selection, processing, and statistical calculations.

## Results

### Basic characteristics of patients

**Fig 2:**
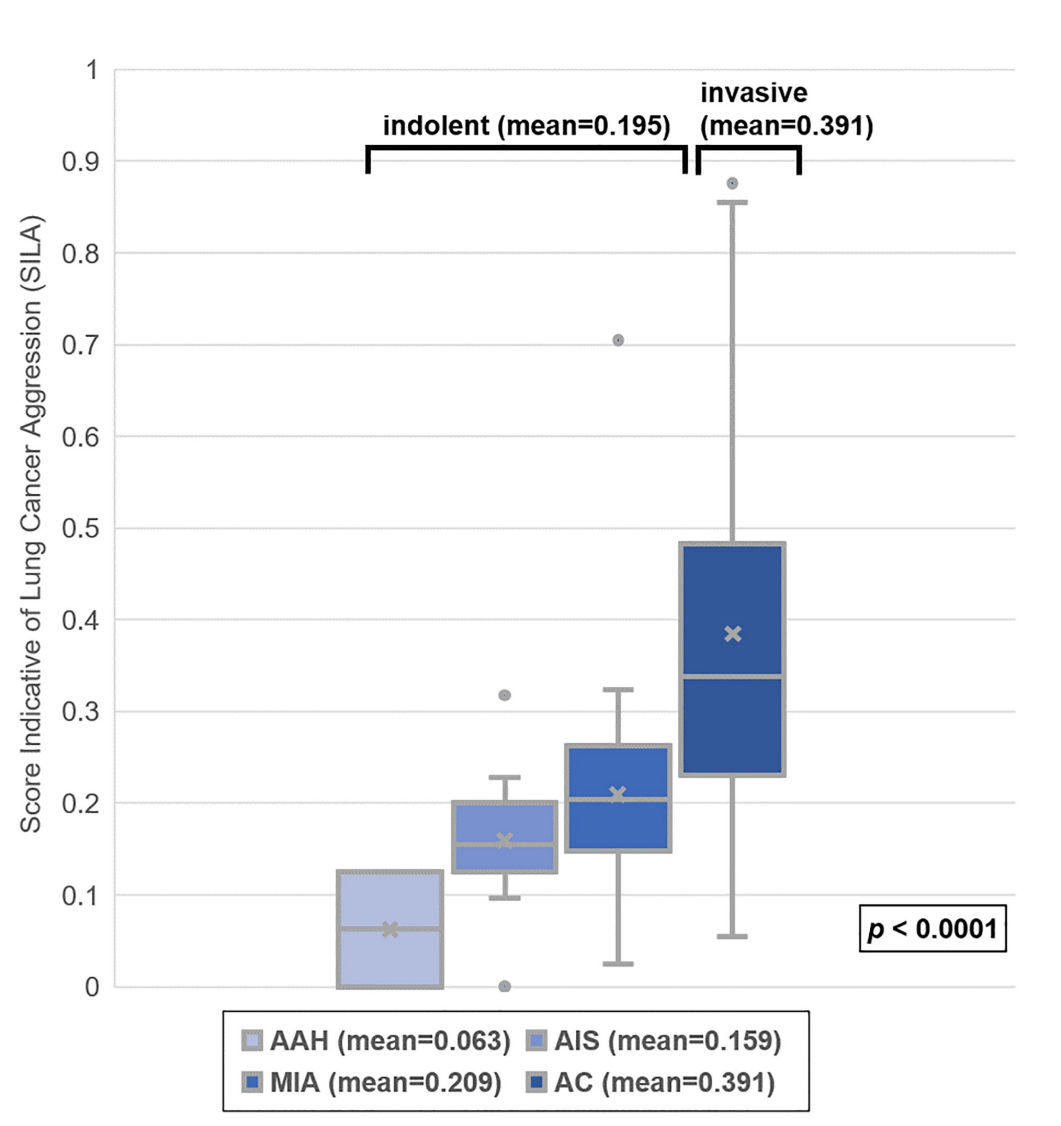
The distribution of score indicative lung cancer aggression (SILA) for each subtype. The mean value and the distribution of the SILA between the indolent and the invasive nodules were statistically significant, and also among the atypical adenomatous hyperplasia (AAH), the adenocarcinomas *in situ* (AIS), and the minimally invasive adenocarcinoma (MIA) were distinct.

Detailed demographic information of patients considered for this study is provided in Table 2. Of the 360 patients, the mean age was 63 ± 9.9 years, and 223 (61.9%) of the total patients were female. The median slice thickness of CT images was 1.25 (0.675–5) mm, and the mean time difference between the operation and CT scan was 26 ± 23.7 d. Of the total, 19 patients had two or more GGNs. The numbers of indolent and invasive based on pathology reports were as follows: 65 (17.1%) indolent lesions, which included 2 (0.5%) AAH, 13 (3.4%) AIS, and 50 (13.2%) MIA, and 315 (82.9%) invasive lesions. The distribution of SILA for the two groups was statistically significant, and the mean SILAs for indolent and invasive were 0.195 ± 0.124 and 0.391 ± 0.208, respectively (*p* < 0.0001, Fig 2). Because the ratios of indolent and invasive were imbalanced, CANARY verification was performed on two datasets: a whole dataset and an equalized dataset.

**Table 2:** Demographic information of 360 patients with 380 ground-glass opacity nodules.

Clinical Characteristics	N (%)
Gender	
Female	223 (61.9%)
Male	137 (39.1%)
Age (years: mean ± SD)	63 ± 9.9
Slice Thickness (mm: median [range])	1.25 (0.675–5)
Interval Between CT to Operation	
days: mean ± SD	26.0 ± 23.7
Smoking Status (pack year: mean ± SD)	
never	266 (73.9%)
former	68 (18.9%) / 28.1 ± 15.4
current	26 (7.22%) / 28.8 ± 21.9
EGFR Mutation	
mutation	127 (35.3%)
wild type	230 (63.9%)
not tested	3 (0.8%)
Type of Lesion	
Pure	80 (21.1%)
Mixed	290 (76.3%)
solid area ≤ 5mm	59 (15.5%)
solid area > 5mm	231 (60.8%)
Solid	10 (0.03%)
Size of Lesion (total / consolidation, cm)	
Pure	1.59 ± 0.68 / 0.00 ± 0.00
Mixed	2.58 ± 1.23 / 1.44 ± 1.10
solid area ≤ 5mm	1.76 ± 0.51 / 0.30 ± 0.13
solid area > 5mm	2.80 ± 1.27 / 1.74 ± 1.05
Solid	3.14 ± 1.98 / 3.14 ± 1.98
Stage^a^ (AJCC 8^th^)	
0	12 (3.2%)
IA	331 (87.1%)
IB	31 (8.2%)
IIA–IIIA	6 (1.6%)
Adenocarcinoma Subtype	
Invasive Adenocarcinoma	315 (82.9%)
Indolent	65 (17.1%)
Minimally Invasive Adenocarcinoma	50 (13.2%)
Adenocarcinoma *In Situ*	13 (3.4%)
Atypical Adenomatous Hyperplasia	2 (0.5%)

SD; standard deviation, CT; computed tomography, AJCC; American Joint Committee on Cancer

^a^The stage described above is for each GGNs, two or more GGN lesions from a single patient were described separately.

### Validation of CANARY in the whole dataset

**Fig 3:**
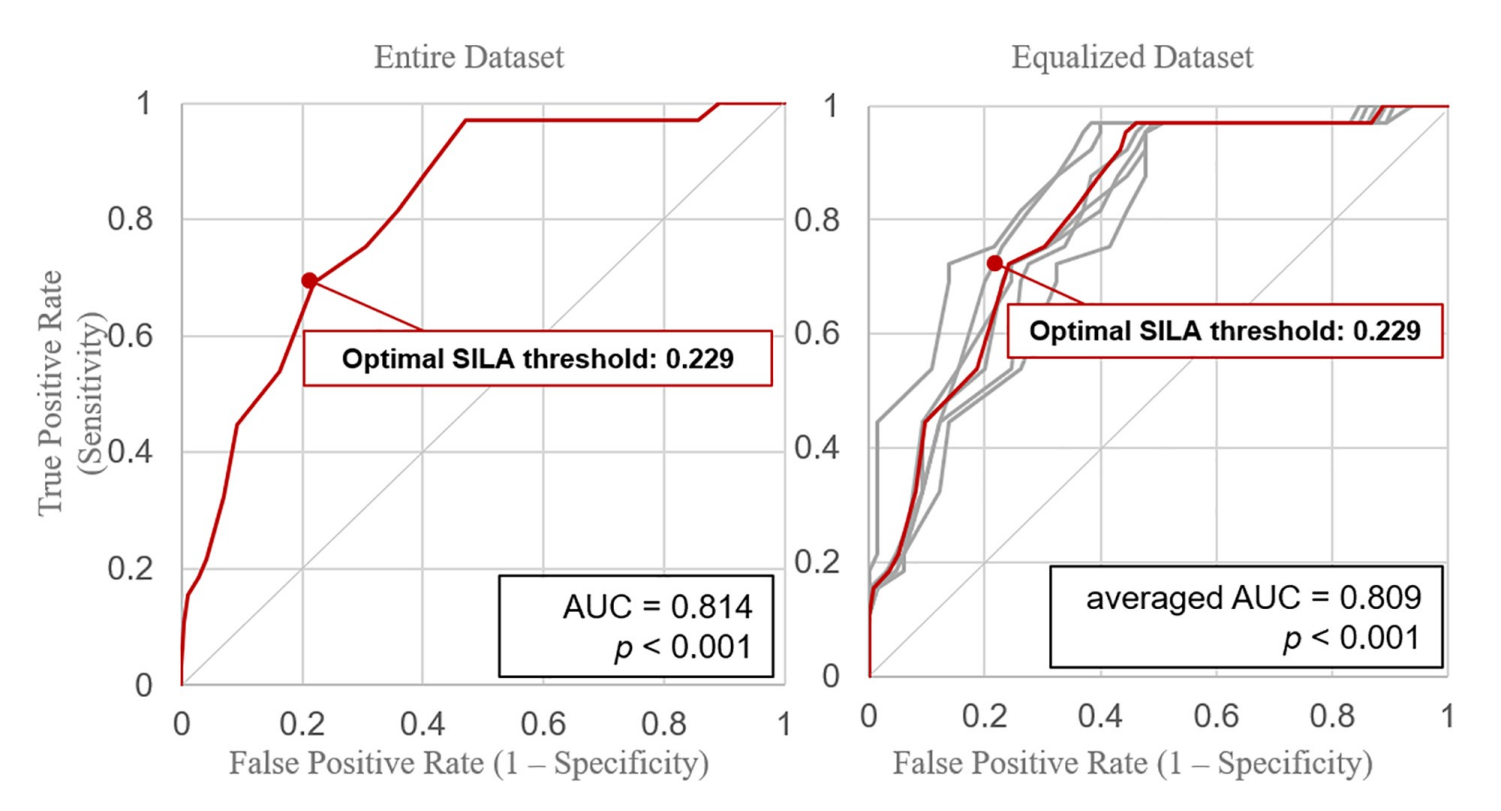
The Receiver Operating Characteristic (ROC) curves of distinguishing indolent and invasive nodules. (A) ROC curve by following Score Indicative of Lung Cancer Aggression (SILA) as thresholds by using the entire dataset. (B) ROC curves of six arbitrarily selected datasets and the macro-averaged curve for evenly distribution of indolent and invasive nodules.

The CANARY analysis results of the entire dataset with uneven distribution for the two classified groups are as follows. To examine the performance of CANARY for distinguishing between indolent and invasive, a ROC curve was drawn with SILA as the threshold (Fig 3). The AUC was calculated as 0.814, and the optimal cutoff SILA was 0.229. Furthermore, at the same SILA, the accuracy, macro F1-score, nMCC, nBM, and GM were recorded as 0.761, 0.675, 0.699, 0.746, and 0.745, respectively. Compared to the results published by the CANARY development group, each of the scores was relatively low (*U* = 2.00, *p* = 0.0131, Table 3).

**Table 3:** Values from each indicator from entire dataset, equalized dataset of this study, and the previous study.

	optimal SILA	AUC	Accuracy	Macro F1-score	nMCC	nBM	GM	*U*	*p-value*
		[0, 1]	[0, 1]	[0, 1]	[0, 1]	[0, 1]	[0, 1]		
Mayo Clinic [4, 9]	0.338	0.912	0.942	0.852	0.858	0.812	0.792		
Entire Dataset	0.229	0.814	0.761	0.675	0.699	0.746	0.745	2.00	0.0131
Equalized Dataset	0.229	0.809	0.741	0.741	0.741	0.741	0.741	1.00	0.0083

AUC; area under the curve, SILA; score indicative lung cancer aggression, nMCC; normalized Matthews correlation coefficient, nBM; normalized bookmaker informedness. GM; geometric mean

### Validation of CANARY in the equalized dataset

Generally, data types are artificially balanced before training or validation of the machine learning clustering model by resampling the data [17]. Because the cases of indolent and invasive lesions were imbalanced, we down-sampled the entire dataset into six randomized and equally distributed datasets, and measured the evaluation scores of each. Both macro and micro average AUC were recorded as 0.809 from six under-sampled datasets that were randomly extracted to ensure that the two classes were distributed evenly (Fig 3), and 0.229 was used as the optimal cutoff SILA. The accuracy, macro F1-score, nMCC, nBM, and GM all had a score of 0.741 at a SILA of 0.229. All of the obtained scores were lower than those of the CANARY development group (*U* = 1.00, *p* = 0.0083, Table 3).

## Discussion

In this study, we evaluated the versatility of CANARY by applying Korean patients. CANARY has been developed through constant validation on various users and institutions, but still has a limitation that it has only been verified in American and European patients [4–9]. This study is of great significance in that it was able to validation CANARY on Asian patients for the first time. Previous studies related to the development and verification of CANARY are summarized in Table 4.

**Table 4:** Previous studies related to CANARY development and verification.

Year	Study	Research group	Dataset	Key point	Performance
2013	Maldonado *et al*. [4]	Mayo Clinic	140	Pilot study	• Categorize “aggressive” or “indolent”: Sensitivity 98.7%, DSC 0.912
				9 exemplars by unsupervised clustering	
2014	Raghunath *et al*. [4]	Mayo Clinic	264	3 groups: G,I,P	• Cohort (Stage I nodules) into three subgroups and compare 5-yr DFS: Good 100%, Intermediate 72.7%, Poor 51.4%
				DFS analysis	
2015	Maldonado *et al*. [5]	Mayo Clinic	294	NLST validation	• CANARY risk groups hazard ratio: Good vs. Intermediate 0.12 & Good vs. Poor 0.09
2017	Clay *et al*. [6]	Mayo Clinic	118	EGFR mutation validation	• Discriminate EGFR mutation (AUC 0.87)
2017	Nemec *et al*. [9]	Mayo Clinic & VUMC	64	Risk stratification	• Component of ≤45% ‘Good’ risk group was associated with histological invasiveness
2018	Nakajima *et al*. [7]	Mayo Clinic	95	Inter- & intra- observer validation	• Inter-observer variability (ICC): VUMC 0.828, Mayo Clinic 0.852
					• Segmentation DSC: VUMC 0.793, Mayo Clinic 0.812
2019	Varghese *et al*. [9]	Mayo Clinic	237	SILA proposed	• Differentiate between “indolent” and “invasive” by using SILA (AUC 0.912)
					• KM survival analysis (stage 1 only); SILA of 0.338 divided the indolent and invasive

DSC; Dice Similarity Coefficient, DFS; Disease-free Survival, NLST; National Lung Screening Trial, VUMC; Vanderbilt University Medical Center, EGFR; Epidermal Growth Factor Receptor, AUC; Area Under the Curve, ICC; Intra-class Correlation Coefficient, SILA; Score Indicative of Lung Cancer Aggression, KM; Kaplan-Meier

When comparing the performance of specific diagnostic tools, the ROC curve and AUC are usually applied. The AUC measured by CANARY in the patients in this study was compared to that reported previously; the AUC of CANARY in data from the Mayo Clinic and that in our dataset were 0.912 and 0.814, respectively [9], and both scores can be regarded as excellent [18]. In addition to AUC, the various evaluation indicators from the confusion matrix were compared between the current study and that of Maldonado et al. (n = 86; indolent = 11, invasive = 75) [4]. This study included a relatively high proportion of false negatives (1 out of 86 from Maldonado’s compared to 73 out of 380 from this study), which resulted in relatively lower scores with an accuracy of 0.181 and macro F1-score of 0.177. However, these basic and widely used metrics have limitations by definition. First, accuracy is inappropriate for imbalanced data because it tends to provide a large advantage to a class containing a large number [19, 20]. Second, even though the F1-score was calibrated to a macro F1-score to overcome disregarding the samples that were correctly classified as negative, the macro F1-score was still biased in the procedure [21]. To overcome these problems, some studies have suggested alternative methods [14, 22]. Among the proposed indicators, nMCC, nBM, and GM have been applied to the confusion matrices from both studies. As a result, the difference in nMCC, nBM, and GM between the two institutions was 0.159, 0.066, and 0.047, respectively, with all the lower values from the current study. The inconsistencies in the scores were relatively smaller in BM and GM, while the differences in accuracy, macro F1-score, and MCC were significantly larger.

Meanwhile, the “relatively objective” performance scores of CANARY can be confirmed from the results of the accuracy or the F1-score from the equalized dataset and the results of the BM or the GM from the entire dataset. As shown in Table 3, the results of our study were approximately in the mid-0.7s, while Maldonado’s results were between the high-0.7s and low-0.8s. From the conventional method conducted by the radiologists, Ye et al. [23] reported that the inter-observer kappa (κ)-value was 0.703 and the intra-observer κ-values were 0.867 and 0.783 for the two radiologists who distinguished between sub-solid and pure-solid lung nodules. In addition, in Maldonado’s study, two thoracic radiologists scored a κ-value of 0.49 in classifying indolent and invasive. Compared to the kappa scores above, it can be observed that it may be clinically useful if the performance indexes of CANARY are slightly supplemented.

There were some differences between the two institutions for several indicators. Apart from the aforementioned results, there was an obvious difference in the optimal cutoff SILA between the two institutions, which was 0.338 in the previous study and 0.229 in ours [9]. There are several reasons for this difference in the optimal cutoff. First, the methods the two institutions used to set the optimal SILA threshold were not identical. The cutoff from the Mayo Clinic study was based on survival rates, while ours was based on pathology reports. However, it is well known that pathologic invasiveness is highly correlated to survival rate [24], so it could be considered that the two approaches were unlikely to affect the cutoff difference. Second, the CANARY observers were different. A previous study [7] showed that even for the same data, the CANARY analysis results were slightly different according to the observer, which might be due to the absence of auto-segmentation. Above all, however, the differences between patients, such as nationality and race, in the datasets used by the two institutions seem to be the principal cause of the discrepancy in the optimal threshold. In addition, there was also a difference in the stage distribution of the cohorts used in the studies of the two institutions. The cohort used in our study was 87, 8, and 2% in stage IA, IB, and higher stages respectively, while the study by Varghese et al, reported 71, 12, and 17% in each stage [9]. The cohort from our study also included adenocarcinoma in situ lesions. In the cohort of both studies, overall, the patients used in this study were in earlier stages, and this difference seemed to have contributed to some extent in lowering the overall cutoff SILA. CANARY was developed based on a machine learning algorithm, and some studies have indicated that if the data used for machine learning training is skewed toward a specific country or race, the algorithm may not be suitable for application to other countries or races. For instance, Gianfrancesco et al. mentioned the potential biases in interpreting medical records and suggested some solutions to minimize overfitting or over-reliance on the model [25]. Furthermore, Popejoy and Fullerton pointed out that the excessive concentration of European data used in genome-wide association studies (GWAS) has resulted in an under-representation of other racial characteristics [26].

As mentioned in the previous paragraph, the difference in datasets used by the two institutions was not the only independent variable, and this is a potential limitation of this study. Moreover, further research is needed to find the cause of the discrepancy in optimal SILA for two institutions. However, our study identified an important component of machine learning algorithms, by demonstrating that the optimal SILA threshold could be different depending on the institution or the race or ethnicity of the patients. Based on our study, we can assume that when a machine learning algorithm based on a specific country or race is applied to other countries or races, the algorithm has to be validated in the new patient set, and the cutoff value has to be adjusted. By setting a customized cutoff, CANARY is potentially clinically beneficial to surgeons with “customized robustness.” In addition, by applying various indicators to verify the performance of CANARY, it was possible to develop a guide for the use of appropriate indicators when evaluating the performance of binary classification models, especially in medical research where unbalanced data types are frequently used.

In conclusion, our study showed that CANARY provided reliable performance in distinguishing between indolent and invasive nodules from the chest CT images of Korean patients, and it could be helpful in the clinic. However, the optimal cutoff value of SILA for differentiation was different from the initial suggested value, and the reasons for these differences must be studied further. In addition, by incorporating a similar approach of CANARY with the emerging automated machine learning method to increase the efficiency and the internet of things technology to simplify the process [26, 28], the research could be extended to other carcinomas of the lung or even to other organs, and related research is underway.
